# Raw milk from individual teats with an optimal teat-end score has lower spore levels compared with teats with a suboptimal teat-end score

**DOI:** 10.3168/jdsc.2025-0802

**Published:** 2025-09-17

**Authors:** Aljoša Trmčić, Rachel L. Evanowski, Sriya Sunil, Martin Wiedmann, Nicole H. Martin

**Affiliations:** Milk Quality Improvement Program, Department of Food Science, Cornell University, Ithaca, NY 14853

## Abstract

Udder health in dairy cows is routinely monitored (for example, through determination of SCC) to help identify and control mastitis and other infectious diseases. Another parameter used to both assess udder health and milking machine performance is the teat-end condition, typically assessed at the herd level using a 4-point scoring system. Recently, the teat-end score has been suggested as a factor associated with the levels of bacterial spores in raw milk. Spores of cold-tolerant sporeforming bacteria present in raw milk can survive pasteurization and contribute to the spoilage of fluid milk. Therefore, the objective of this study was to determine whether the condition of individual teat-ends (specifically, optimal versus suboptimal teat-end scores) affects the spore levels in raw milk obtained from these teats. A total of 102 raw milk samples were collected from 102 individual teats from cows on a single dairy farm, and tested for mesophilic spores, with 51 samples each from teats scored as either 1 (optimal) or 4 (suboptimal). A significantly lower mesophilic spore count was found in the raw milk samples collected from teats with a teat-end score of 1 compared with samples collected from teats with score of 4. The observed difference in mesophilic spore counts suggests that maintaining optimal teat-end condition through good udder health and milking machine management may help reduce spore levels in raw milk. Although the observed difference in mesophilic spore counts is expected to result in minimal improvement of fluid milk shelf-life and other quality parameters, interventions targeting teat-end status or farm management practices that aim to improve the same can still be an important incremental contributor to the overall improvement of these parameters as part of a comprehensive, multipronged approach to raw milk quality management.

## Highlights

• Spore levels were determined in raw milk from optimal and suboptimal teat-ends.
• Spore levels were lower in raw milk obtained from teats with optimal teat-ends.
• Lower spore level is expected to minimally increase the pasteurized milk shelf-life.
• Teat-end condition can contribute to incremental improvement of raw milk quality.

## Graphical Abstract

**Figure:**
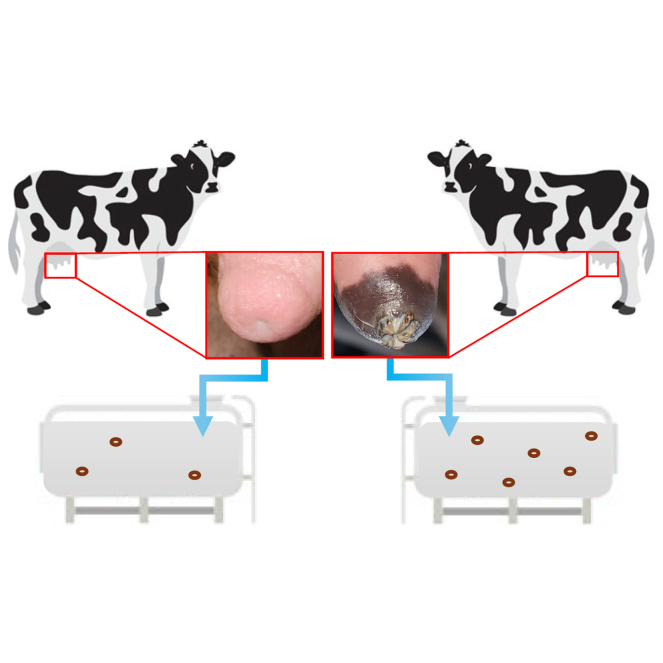
**Summary:** Spores of sporeforming bacteria in raw milk can survive pasteurization and cause spoilage of fluid milk. To assess the previously suggested link between teat-end condition and spore levels, we determined the mesophilic spore count in a total of 102 raw milk samples obtained from an equal number of teats with optimal (score 1) and suboptimal (score 4) teat-end scores. A statistically relevant difference in mesophilic spore count was observed between the 2 groups. The observed difference is expected to result in minimal extension of pasteurized fluid milk shelf-life. Although improving teat-end condition alone may not substantially extend shelf-life, it can still contribute to incremental improvement of overall raw milk quality as part of a broader quality management strategy. Created in Canva.

Sporeforming bacteria are a major microbial concern in raw milk due to their ability to form heat-resistant spores that can survive pasteurization. Subsequent germination and growth in dairy products after pasteurization can lead to spoilage and considerable economic losses (Huck et al., 2007; Ranieri and Boor, 2009; Buzby et al., 2014). For example, the maximum shelf-life that pasteurized fluid milk can achieve is commonly limited by the presence of sporeformers that are predominantly introduced through raw milk before pasteurization. Some sporeformers (e.g., *Paenibacillus* spp.) are of particular concern because, even though they are mesophilic microorganisms that prefer growth at temperatures between 21°C and 37°C, they can also grow at refrigeration temperatures and reach concentrations in pasteurized milk that cause off-odors or off-flavors (Huck et al., 2007; Trmčić et al., 2015). Hence, controlling the presence and levels of spores in raw milk at the farm level is critical to ensuring product quality and extending the shelf-life of pasteurized fluid milk at the processor, retail, and consumer level.

A range of farm-level management factors has been associated with spore levels in raw milk. These include housing practices, hygiene practices during milking, type of bedding used, and the cleanliness of the udder and teats (Masiello et al., 2017; Martin et al., 2019; Murphy et al., 2019). For example, higher proportions of cows with dirty udders and dirty housing areas have been associated with elevated mesophilic spores in bulk tank milk (Murphy et al., 2019). Additionally, a recent study of certified organic dairy farms in the United States associated reduced spore levels in bulk tank milk with the practice of removing udder hair through clipping or singeing, which can substantially contribute to maintaining clean udders (Wasserlauf-Pepper et al., 2025).

The key points of contact between the environment and milk are the teat-ends. Teat-ends, when compromised due to poor milking practices (e.g., overmilking) or poor hygiene, may accumulate keratin or develop rough surfaces that support bacterial attachment and transfer into raw milk during milking (Zecconi et al., 2002; Edwards et al., 2013). Teat-end scoring provides a standardized way to assess the severity of hyperkeratosis or other physical changes of the teat opening (Ruegg and Reinemann, 2005), but this type of assessment has, to our knowledge, been underutilized in studies on the relationship between farm-level practices and microbial composition of raw milk. In contrast, udder health is commonly assessed through SCC and remains a strong focus of both dairy producers and researchers. The SCC is strongly influenced by the presence of udder inflammation and is widely used to monitor the occurrence of mastitis, even in the absence of clinical symptoms (Dohoo and Leslie, 1991). However, a recent systematic review on this topic showed a limited association between teat-end score/presence of hyperkeratosis and SCC or udder inflammation (Pantoja et al., 2020). This is consistent with day-to-day fluctuations in SCC for a single cow that cannot be explained by changes in the condition of teat-ends (Kelton, 2017). Although SCC can provide valuable insight into udder inflammation and infection status, it might not directly capture the potential for environmental contamination of teat-ends with spores.

While one study conducted at 5 separate dairy farms showed that a higher proportion of very rough teat-ends in a milking herds was associated with significantly higher thermophilic spore counts in bulk tank milk (Evanowski et al., 2020), the relationship between physical teat-end condition and spore levels in raw milk has not been extensively studied to date. Given that teat-ends represent a direct route for environmental microbes to enter the milk, and that various hygiene and housing factors influence both teat-end condition and spore levels, improving the condition of teat-ends may be a potential target for future interventions. The objective of this study was to evaluate whether physical teat-end condition, assessed using a standardized scoring system (Ruegg and Reinemann, 2005), is associated with levels of spores in bulk tank raw milk.

The calculation of raw milk sample size for this study consisted of 2 separate steps: (1) predict the initial spore concentration difference needed between raw milk obtained from teats with optimal (score 1) and suboptimal (score 4) teat-ends, and (2) calculate the sample size needed to detect this spore concentration difference in raw milk (Equation 1; Dohoo et al., 2009). A modified Monte Carlo simulation model, described by Evanowski et al. (2023), was used to predict the required difference in spore concentrations that would result in a 2-d increase in shelf-life, which is assumed to represent a meaningful change; this difference in spore concentration was subsequently used as the basis of the sample size calculation, by assuming that it represents a target, theoretical difference in the spore concentration of milk from teat-ends with score 1 and those with score 4. Specifically, the mean spore concentration of the fluid milk on d 0 of shelf-life, in the modified Monte Carlo simulation, was used as the input for *μ*_1_ (i.e., −0.72 log_10_ cfu/mL) in Equation 1; this was assumed to represent the spore concentration in milk from teats scored 4. The Monte Carlo simulation was subsequently used to back-calculate the mean spore concentration that would result in the fluid milk units reaching their maximum concentration 2 d later than observed in the baseline simulation; this value was used as the input for *μ*_2_ (i.e., −1.32 log_10_ cfu/mL) and was assumed to be the spore concentration in milk from teats with score 1. For *σ* in Equation 1, we selected the standard deviation of *μ*_2_ (i.e., 1.09 log_10_ cfu/mL) as it was greater than the standard deviation of *μ*_1_ (i.e., 0.99 log_10_ cfu/mL). As a larger *σ* results in a larger sample size, we opted to use the larger standard deviation to obtain a conservative (i.e., higher) sample size. The α (*Z_a_*) and power (*Z_b_*) for the sample size calculation were 5% and 80%, respectively:

[1] $$n=2\;\left(\frac{{\left(Z_{a\;}-Z_{b}\right)}^{2}\sigma^{2}}{{\left(\mu_{1}-\;\mu_{2}\right)}^{2}}\right)$$

A single dairy farm from the northeast United States was recruited to participate in this study. The recruited farm used conventional management practices (i.e., “nonorganic”) and milked approximately 1,300 cows in a parlor 3 times per day. Raw milk samples were collected during one milking shift in December and included 51 raw milk samples from teats with a score of 1, and 51 samples from teats with a score of 4. Scores were assigned by trained personnel using the University of Wisconsin Milk Quality “Teat Condition Scoring Chart” (Ruegg and Reinemann, 2005). This scoring chart scores the teat-ends on a 1 to 4 scale, with 1 being the best possible score and 4 being the worst. Teat-ends with a score of 1 were in optimal condition and had no ring around the teat canal, whereas teat-ends with a score of 4 had the highest level of damage or keratosis and had a very rough ring of keratin that extended more than 3 mm with a flowered appearance (Figure 1). The teat-ends were dipped with an iodine-based predip, forestripped, and wiped by milking staff before sample collection. The milk samples (∼60 mL per teat) were collected in a sterile vial before unit attachment. Samples were stored in coolers packed with ice and transported to the Milk Quality Improvement Program laboratory (Ithaca, NY) within 8 h and were stored upon arrival at −20°C for up to 1 wk; data from previous studies indicate that the ability of dairy-associated spores to germinate and grow is not affected by frozen storage (Lee et al., 2024). Frozen samples were defrosted during 24 h of incubation at 6°C before microbiological analyses. Raw milk samples were shaken in accordance with Standard Methods for the Examination of Dairy Products (Martin et al., 2024) and transferred into individual sterile screw-capped glass tubes before heat treatment. Samples were heat-treated at 80°C for 12 min in accordance with Standard Methods for the Examination of Dairy Products to eliminate vegetative cells and initiate germination of bacterial spores (Boor and Martin, 2024). Samples were cooled on ice until they reached 6°C. All samples were pour-plated in standard methods agar (**SMA**); a total of 10 mL of each sample was plated by pour-plating 1 mL of sample in each of 10 separate SMA plates. Plates were incubated at 32°C for 48 h before enumerating the colonies, representing an aerobic mesophilic spore count (**MSC**). Enumeration was performed on an automated colony counter (Q-count, Advanced Instruments, Norwood, MA) according to the manufacturer's instructions. Testing raw milk samples for cold-tolerant spore counts (psychrotolerant spore count; **PSC**) requires expensive and labor-intensive testing techniques that can fail to produce sufficient quantitative results to perform statistical analysis and draw meaningful conclusions (Evanowski et al., 2020); for this reason the raw milk samples were tested for MSC and results were used as a proxy for the PSC differences expected in the raw milk samples.

**Figure 1:**
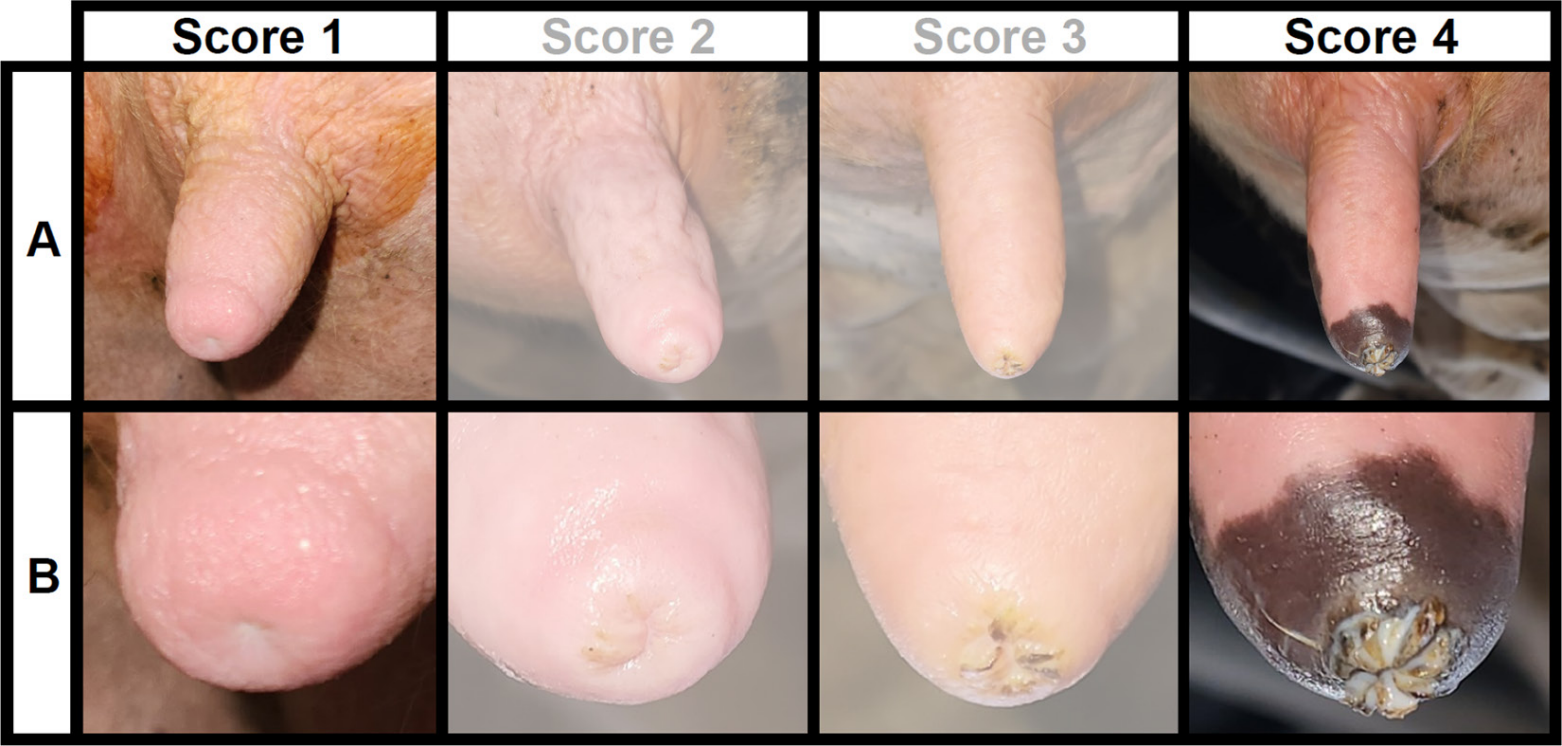
Representative lactating dairy cow teats (A) and teat-ends (B) with teat-end scores 1 through 4 observed among milking cows of the participating dairy farm. Although examples of all 4 types of teat-ends are presented, raw milk for this study was only collected and evaluated for teats with teat-end scores 1 and 4, and raw milk from teats with teat-end scores 2 and 3 were not included in this study. Teat-ends with score 1 were in optimal condition without a ring around the teat canal, and teat-ends with score 4 were in the most suboptimal condition, with the highest level of damage or keratosis, and had a very rough ring of keratin that extended more than 3 mm with a flowered appearance.

The MSC data were collected and managed in Excel (version 2408, Microsoft Corp., Redmond, WA). The MSC data were log-transformed before analysis using R statistical software together with the R stats package (version 4.0.2; R Core Team, 2020). A one-sided Wilcoxon rank sum test was performed on the MSC data to evaluate whether MSC in raw milk from teats with a teat-end score of 1 was significantly lower than in raw milk from teats with a teat-end score of 4; the significant difference was set at *P* ≤ 0.05.

The MSC in the 51 raw milk samples obtained from teats with teat-end score of 1 ranged from −1.00 to 0.91 log_10_ cfu/mL (Figure 2). The median concentration in these samples was 0 log_10_ cfu/mL, with an interquartile range of −0.26 to 0.28 log_10_ cfu/mL. The MSC in the 51 raw milk samples obtained from teats with teat-end score of 4 ranged from −0.30 to 1.23 log_10_ cfu/mL. The median concentration in these samples was 0.32 log_10_ cfu/mL, with an interquartile range of 0.16 to 0.46 log_10_ cfu/mL. The upper and lower outliers of MSC found in raw milk samples collected from teats with a teat-end score of 4 were 1.23 and −0.30 log_10_ cfu/mL, respectively. Comparing the MSC in raw milk samples collected from teats with the 2 different teat-end scores, using a one-sided Wilcoxon rank sum test, showed a significantly lower count in the samples collected from teats with a teat-end score of 1 (*P* < 0.0001). Although obtained data on MSC were not normally distributed, for the purpose of comparison with past and future studies, the mean and standard deviation of the spore concentration in the milk samples, by teat-end score, were (1) −0.05 ± 0.47 log_10_ cfu/mL for raw milk samples from teat-ends with score 1, and (2) 0.35 ± 0.27 log_10_ cfu/mL for raw milk obtained from teats with score 4.

**Figure 2:**
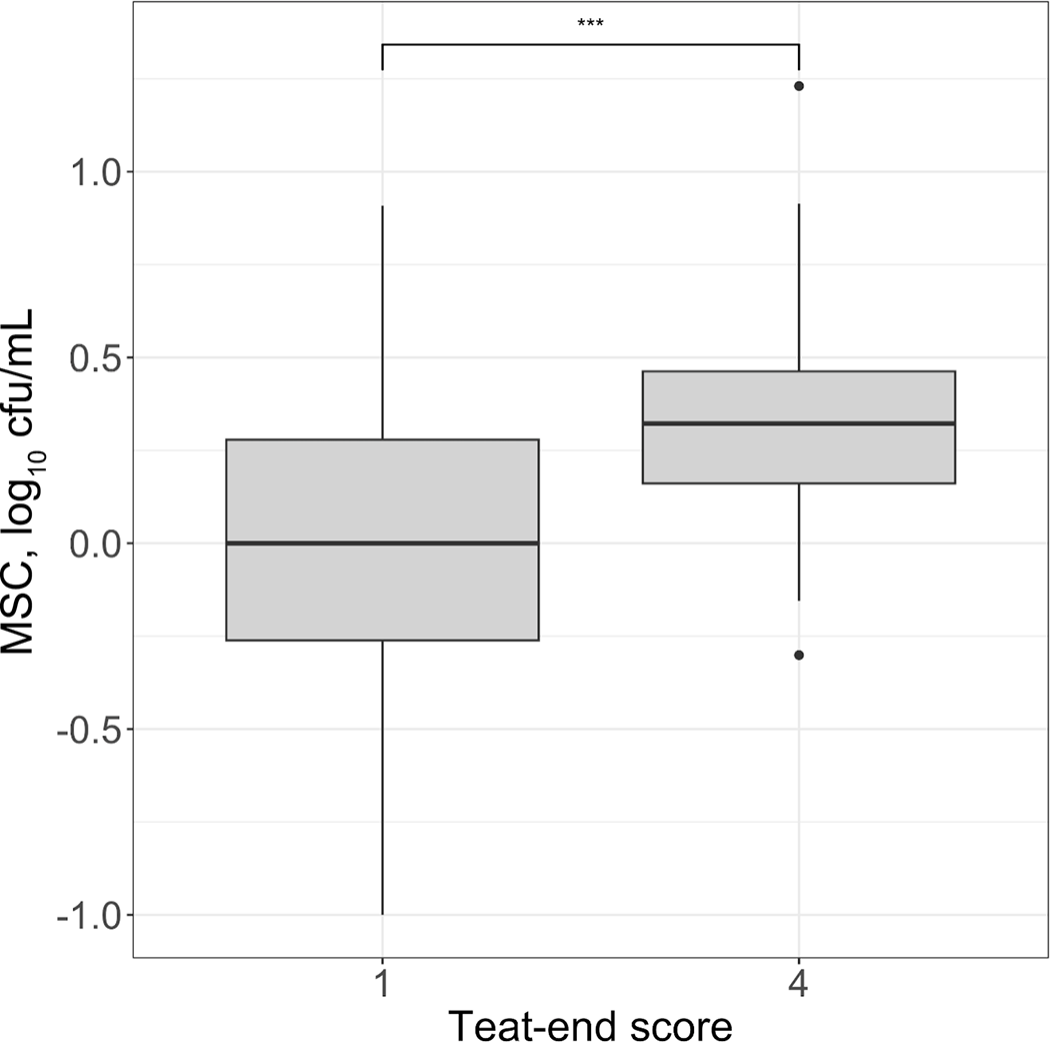
Aerobic mesophilic spore count (MSC) in raw milk samples collected from 102 individual teats with optimal (1) and suboptimal (4) teat-end scores. The bold horizontal lines within the boxplots represent median MSC values; ends of each box represent the first and third quartiles; whiskers represent minimum and maximum values, excluding outliers, which are represented as the 2 individual black points. The top bracket (***) represents a significant difference (*P* < 0.0001) determined using a one-sided Wilcoxon rank sum test.

Previous research has evaluated the bulk tank milk spore counts and determined a similar concentration range of MSC. For example, a mean MSC of 0.26 log_10_ cfu/mL was reported in one study where bulk tank raw milk samples from 190 farms in 18 US states were tested (Murphy et al., 2019). In a study by Martin et al. (2019), where 34 samples of bulk tank raw milk from 17 New York dairy farms were tested, the reported mean MSC was 0.50 log_10_ cfu/mL, and slightly lower (0.42 log_10_ cfu/mL) when raw milk samples from individual cows on these farms were tested. Another study, conducted on 5 different dairy farms in New York, tested a total of 355 bulk tank milk samples and reported a mean MSC of 0.30 log_10_ cfu/mL (Evanowski et al., 2020). The MSC and other bulk tank raw milk spore levels reported in the current and other studies performed by our group remain low; however, even these low spore levels can survive the pasteurization process and go on to grow and cause spoilage of fluid milk and other dairy products, making them an important factor to control in raw milk. Currently, there are no regulatory limits or broadly accepted industry guidelines in the United States on spore levels in raw milk (Martin et al., 2023); instead, the monitoring of spore counts in raw milk is primarily performed by best-in-class dairy processors that want to extend fluid milk shelf-life beyond 21 d that are typically achieved by these processors.

In the study by Evanowski et al. (2020), an intervention focusing on teat-end cleaning during milking preparation was evaluated, and demonstrated a reduction in the mean bulk tank milk MSC from 0.30 to 0.20 log_10_ cfu/mL after the intervention. In the same study, the effect of teat-end condition on spore levels in bulk tank raw milk was evaluated using a mixed-effects linear regression analysis, demonstrating that the proportion of teat-ends in the milking herd with a score of 4 (i.e., very rough teat-ends) was associated with elevated thermophilic spore counts (**TSC**) in the bulk tank milk. Although the same association was not demonstrated for MSC, the same statistical analysis revealed that both MSC and TSC were significantly reduced (*P* ≤ 0.02) in bulk tank milk during milking shifts where research staff were present compared with shifts where research staff were not present. The research group hypothesized that the presence of research staff affected the actions of the parlor employees and how thoroughly they performed teat-end cleaning during udder preparation. This is relevant because one of the previous studies showed that differences in udder health parameters (e.g., SCC, presence of pathogens, and incidence of clinical mastitis) between udders with higher or lower teat-end scores are only observed when postmilking disinfection of udders and cleaning of milking equipment are not performed or performed poorly (Gleeson et al., 2004). This indicates that teats with suboptimal teat-end conditions are more open to the intrusion of microbial contaminants into the teat canal, including spores; however, this intrusion cannot occur if microbial contaminants are appropriately removed or inactivated, regardless of the teat-end condition. This also indicates that several different factors, not a single factor, can be associated with spore levels in bulk tank raw milk and affect the shelf-life and quality of the final dairy product.

In the current study we found a significant difference in MSC (*P* < 0.0001) counts in raw milk obtained from teats with teat-end scores 1 and 4 (i.e., median of 0 and 0.32 log_10_ cfu/mL, respectively), but this minimal difference is not expected to result in a meaningful difference in fluid milk shelf-life and other quality parameters in isolation. In a related study investigating the effectiveness of bactofugation for spore removal from raw milk, Griep-Moyer et al. (2022) predicted using a simulation model that a reduction in PSC from −0.97 to −1.90 log_10_ cfu/mL in pasteurized milk would extend the shelf-life by 1 to 2 d. The same study also quantified MSC in those samples, observing a reduction from 0.99 to −0.49 log_10_ cfu/mL with the use of bactofugation. Based on these results, we can assume that the difference in MSC observed in our current study between raw milk obtained from teats with scores 1 and 4 would correspond to PSC differences that would most likely result in less than 1 d extension of pasteurized milk shelf-life. According to expert opinion provided by one large US fluid milk processor (who requested to remain anonymous), extending shelf-life of fluid milk by less than 1 d is not sufficient to reduce the cost of processing and transport by reducing the number of changeovers and number of trucks needed for delivery, or increasing the time and distance the product could be transported. However, while relying on a single intervention (e.g., directing the farm management practices toward improved teat-ends) is not likely to result in meaningful improvement of shelf-life and quality parameters of fluid milk and other dairy products, teat-ends status represents one of the factors that can contribute to incremental improvement of these parameters as part of a comprehensive multipronged approach that includes practices previously shown to reduce spore levels in bulk tank raw milk (e.g., removal of udder hair, or training milking employees to adequately clean teat-ends).

Results of our current study, together with results of other studies performed by our group and other research groups, highlight the importance of several farm factors that are directly and indirectly associated with spore levels in bulk tank raw milk; for example, housing practices, type of bedding used, hygiene practices during milking, removing udder hair through clipping or singeing (or both), and other means of maintaining the cleanliness of the udder and teats. It is therefore necessary to approach bulk tank spore reduction with a comprehensive whole-farm approach that addresses these factors, resulting in incremental and cumulative spore decreases. Farm-level improvements expected from a comprehensive farm management plan must be supported by equally comprehensive dairy processing, transportation, and storage management plans to ultimately achieve a meaningful reduction in the spoilage of final pasteurized dairy products.
